# Impact of social distancing from the COVID-19 pandemic on the immuno-inflammatory response of older adults

**DOI:** 10.1186/s12877-024-04699-7

**Published:** 2024-01-26

**Authors:** Giulia Beletato Nery, Carlos Ariel Rodrigues de Araujo, Giovanna Beatriz da Silva, Helena Bittar, Valéria Pacheco Bordallo, Jônatas B. Amaral, Markus Hardt, Luciana Marti, Alexander Birbrair, Manuel Jimenez, Marta Ferreira Bastos, Luiz Henrique Silva Nali, Priscila Larcher Longo, Gilberto Candido Laurentino, André L. L. Bachi, Debora Heller

**Affiliations:** 1 Post Graduate Program in Dentistry, Cruzeiro Do Sul University, São Paulo, Brazil; 2 Department of Otorhinolaryngology, ENT Lab, Federal University of Sao Paulo (UNIFESP), São Paulo, Brazil; 3 Center for Salivary Diagnostics, The Forsyth Institute, Cambridge, MA USA; 4 Department of Developmental Biology, Harvard School of Dental Medicine, Boston, MA USA; 5 Experimental Research, Hospital Israelita Albert Einstein, São Paulo, Brazil; 6 Department of Dermatology, School of Medicine and Public Health, University of Wisconsin-Madison, Madison, WI USA; 7 Departamento de Didáctica de La Educación Física y Salud, Universidad Internacional de La Rioja, Logroño, Spain; 8 Postgraduate Program in Aging Sciences, São Judas Tadeu University, São Paulo, Brazil; 9 Post-Graduate Program in Health Science, Santo Amaro University (UNISA), Santo Amaro, Brazil; 10 Department of Periodontology, UT Health San Antonio, San Antonio, TX USA

**Keywords:** Mucosal immunity, Saliva, Cytokines, Interferon, Interleukin, SARS-CoV-2

## Abstract

**Background:**

Older adults, as the population considered at increased risk for severe COVID-19, were the most impacted by social isolation. Thus, this study aimed to assess the salivary immune/inflammatory response of older adults before and during the COVID-19 pandemic.

**Methods:**

A cohort of 11 older adults (mean age 66.8 ± 6.1) was followed at three different time points: before (S1) and after 6 (S2) and 20 months (S3) of the beginning of the COVID-19 pandemic in Brazil. Unstimulated saliva samples were obtained to assess the levels of antibodies (secretory IgA, IgG and IgM) by ELISA and cytokines (IL-2, IL-5, IL-6, IL-8 and IL-10, TSLP, IFN-γ, TNF-α) by multiplex analysis. Significant differences were evaluated using the Kruskal–Wallis test with Dunn's post-test.

**Results:**

None volunteer presented periodontal disease or caries. All volunteers received at least two doses of the COVID-19 vaccines after S2 and before S3. A tendency to increase salivary levels of SIgA and IgM at S2 and of IgG at S3 were observed compared to the values found at S1 and S2. Significantly decreased levels of IL-2 and IL-5 were found at S2 and S3 (*p* < 0.001) time points. Lower levels of IFN-γ were found at S2 as compared to the values observed at S1 (*p* < 0.01). A significant decrease in the IFN-γ/IL-10 ratio was found at S2 (*p* < 0.01). When assessing the Th1/Th2 ratios, a significant decrease was found in the IFN-γ/TSLP ratio at S2 (*p* < 0.001) and S3 (*p* < 0.001) when compared to the values at S1. In addition, a significant increase was observed in the TNF-α/IL-5 ratio at S2 (*p* < 0.001) and S3 (*p* < 0.001) in comparison to the values at S1. In a similar way, an increase in the TNF-α/IL-6 ratio (Fig. 5E) was observed at S3 (*p* < 0.001) when compared to the values at S1.

**Conclusions:**

Overall, this study provides valuable insights into the impact of COVID-19-induced social isolation on immune/inflammatory responses in the upper airway mucosa, particularly those present in oral cavity, of older adults. It demonstrates that a controlled shift in Th1 and Th2 immune responses, both during infection and post-vaccination, can create favorable conditions to combat viral infections without exacerbating the immune response or worsening the pathology.

## Background

From 30 January 2020 – 5 May 2023, the World Health Organization (WHO) characterized the global spread of coronavirus disease 2019 (COVID-19) as a pandemic and a public health emergency of international. More than 690 million people have been infected with SARS-CoV-2, which is mainly spread via respiratory droplets and aerosols from infected individuals. Upon binding to epithelial cells in the respiratory tract and the oral cavity, SARS-CoV-2 can invade the host and replicate, triggering a strong immune/inflammatory response in predisposed individuals, known as cytokine storm syndrome. This hyper-inflammatory response has been considered the main cause of death of COVID-19 patients [1].

Importantly, during the COVID-19 pandemic the older adult population was commonly instructed to self-isolate for long periods of time [2] in an attempt to shield the over 60 years-old individuals, and protect the health systems from over-burdening. However, the deleterious effects of social isolation on general health, oral health and overall well-being have been well established, and stressors during crisis have been associated with the triggering of an immune dysfunction characterized by abnormal production of inflammatory cytokines [3]. During the COVID-19 pandemic, several studies explored the impact of social isolation on inflammation context [4]. In fact, prolonged periods of quarantine and social distancing were closely associated with increased levels of stress, anxiety, and loneliness, which, in turn, contributed to elevated inflammation markers [5]. Studies also have indicated that the psychological stressors related to social isolation might elicit a body´s inflammatory response, which potentially affects the immune system [6, 7]. Moreover, it is well-known that chronic inflammation has been implicated in various health conditions, including cardiovascular diseases, frailty, diabetes, and mental health disorders, among others, mainly in aged people [8] these aspects, it is utmost of importance to highlight that the older adults, as the population considered at increased risk for severe COVID-19, were the most impacted by social isolation [9].

Whilst multiple serum biomarkers have been described for monitoring the immune response in older patients [10], we have shown that these biomarkers can also be measured in saliva (Pacheco et al. 2022).

In this context, saliva presents an opportunity for monitoring local adaptive immunity. Recently, our group showed, for the first time, that distinct associations between immunological cytokine profiles can help maintaining the salivary levels of secretory immunoglobulin A (sIgA) in mild COVID-19 cases [11]. Furthermore, it is noteworthy to highlight that the implementation of salivary diagnosis is highly desirable, due to the fact that saliva provides a mean for self-collected, non-invasive, safe, and low-cost biological samples that are suitable to improve health care accessibility [12].

Our fundamental question was to verify whether the social isolation imposed by the COVID-19 pandemic on older adults could be able to trigger an immune dysfunction characterized by abnormal production of different types of cytokines. Based on it, we hypothesized that the intricate interplay among social factors, mental health, and physiological responses during the period of social isolation triggers an inflammatory response, which can be monitored through saliva. Thus, we propose in this study the analysis of salivary immune/inflammatory response of older adults. Specifically, this study assessed inflammatory markers in saliva collected from older adults before and during the COVID-19 pandemic, as well as the multiple other factors that contribute to the oral and systemic health of these individuals.

## Methods

### Study design

The present study was designed as a longitudinal cohort study and was conducted in full accordance with the Helsinki Declaration of 1975, as revised in 2000 and approved by the local Human Research Committee at the Universidade São Judas Tadeu (Protocol 3.373.066). To be enrolled, participants were individually informed about the nature of the proposed study, its risks and benefits, and required to sign informed consent forms.

Concerning the social isolation imposed by the COVID-19 pandemic, all volunteers self-reported, by video calls, alteration in their daily routine, which was mainly associated with the reduction of many activities performed outside their homes during this period, including physical exercises, familiar visits, voluntary work, walk-in parks and shopping malls, among others. However, they reported having strong social networks.

### Participants and data collection

The older adult population who participated in the present study was recruited at the Universidade São Judas Tadeu, Brazil, including 10 older women and 1 older man and presented a mean age of 66.8 ± 6.1 years old. Inclusion criteria included older adults in community-dwellings or living autonomously. Individuals with genetic, musculoskeletal, or uncontrolled endocrine diseases, or who had chronic alcohol consumption or previous use of illicit drugs, or who used anti-inflammatory drugs, even for acute treatments for the past 6 months, were excluded from the study.

In relation to the number of participants enrolled in the present study, it is necessary to mention that those volunteers were initially invited to participate in another study before the pandemic. On this occasion, a sample size calculation was performed to establish a minimum number of participants and by using the G*Power software program, the sample size and statistical power were estimated, based on the Student’s T-test, effect size (0.30) at α-level (0.05), statistical power of 0.95, and two groups with two (pre-pos) measures. Considering a margin of 30% losses or refusal, a minimum of 16 individuals per group were putative. However, when the COVID-19 pandemic began, the previous study was stopped, but the same volunteers were invited to participate in the present study, and after contact with those volunteers, only 11 older adults voluntarily agreed to participate. Therefore, the present study was performed with a convenience sample.

A single calibrated examiner (V.B.P.) was responsible for collecting sociodemographic (sex and age), anthropometric, and clinical characteristics, including periodontal disease and caries. The decayed, missing, and filled teeth (DMF-T) index was used to assess older adults’ accumulated history of dental caries [13]. To assess periodontal health status, clinical parameters including bleeding on probing (BOP) and suppuration (SUP), in addition to the probing depth measurement (PD (mm)) were evaluated by a calibrated periodontist at 6 sites per tooth (mesiobuccal, buccal, distobuccal, mesiolingual, lingual, and distolingual) of all teeth, except for the third molars [14]. Periodontal clinical parameters were evaluated as they reflect potential intraoral sources of inflammation that, once expressed in saliva, could confound the assessment of the impact of social distancing on the immuno-inflammatory response of older adults.

**Fig. 1:**
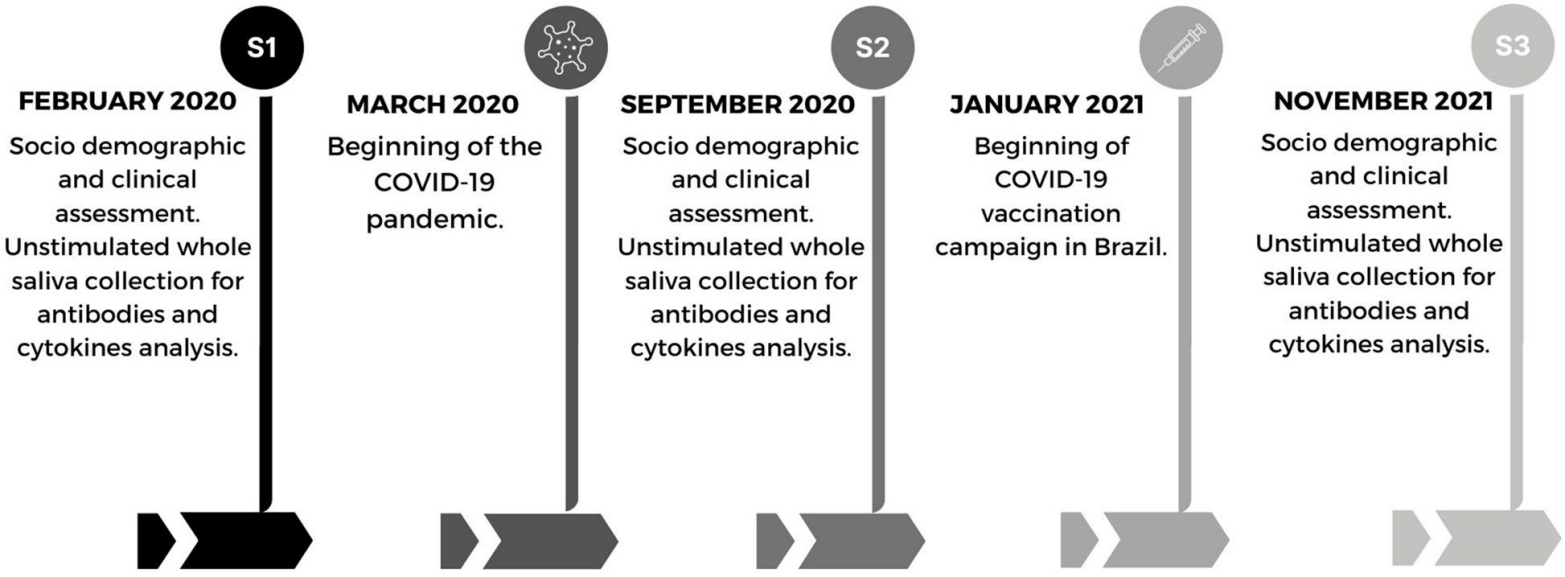
Design of the study: Social demographic, oral and systemic assessments and saliva collections: S1 (Before the COVID-19 pandemic in Brazil and baseline saliva sample collection), S2 (second saliva sample collection after 6 months of the beginning of the pandemic), S3 (third saliva sample collection after 20 months of the beginning of the pandemic)

Study participants were examined at three different time points: before (T1) and after 6 (T2) and 20 months (T3) of the beginning of the COVID-19 pandemic in Brazil (Fig. 1).

### Assessment of respiratory viruses infection by RT-PCR

Naso-oropharyngeal samples were obtained from volunteers who presented any respiratory symptoms and were used to assess the occurrence of infection by respiratory viruses. Total RNA was extracted by using the NUCLISENS® easyMag platform (bioMérieux, Massachusetts, USA), and submitted to the real-time PCR, which was performed on ABI 7300 machine utilizing the AgPath-ID One-Step RT-PCR master mix kit (Applied Biosystems Inc., USA). It was evaluated a panel of respiratory viruses, which included: SARS-CoV-2, Respiratory Syncytial Virus type A and B (RSV-A/B), Human Metapneumovirus (HMPV), Parainfluenzavirus (PIVI-1–4), Adenovirus (AdV), Rhinovirus (RV), Influenza Virus type A and B (Flu- A/B), and seasonal Coronavirus type 1–4 (CoV 229E, CoV OC43, CoV NL63, CoV HKU1).

### Unstimulated whole saliva collection, processing, and storage

Unstimulated whole saliva (UWS) samples were self-collected for antibodies and cytokines analysis as previously described [15]. The samples were stored for no longer than 3 months.

### Specific antibodies assessments for SARS-CoV-2 antigens

Unstimulated saliva samples were obtained to assess the levels of specific antibodies (secretory IgA, IgG, and IgM) for SARS-CoV-2 antigens by an in-house developed ELISA assay as described previously [11]. All analysis were performed in duplicates.

### Cytokines assessment

Salivary cytokine concentrations (Interleukins (IL)-2, -5, -6, -8 and -10, Thymic Stromal lymphopoietin (TSLP), Interferon (IFN)-γ, Tumor Necrosis Factor (TNF)-α and Klotho were assessed by a multiplex assay (LEGENDplex™ bead-based multiplex assays, Biolegend, San Diego, CA, USA), following the manufacturer’s instructions. Each cytokine concentration was calculated using an appropriate standard curve. The multiplex assay linearity was within the 2.4–10,000 pg/mL range and correlation coefficients of all standard curves were between 0.95 to 0.99, while the intra-assay variance coefficients were 3–5% and inter-assay variance coefficients 8–10%. Analysis was carried out by using the BD Accuri™ C6 Plus Flow Cytometer (BD Biosciences San Jose. CA. USA) and the data obtained were analyzed with LEGENDPlex™ V8.0 software (Biolegend). All analysis were performed in duplicates.

### Statistical analyses

All analyzes were performed using the SPSS statistical program version 19.0 (IBM, São Paulo, Brazil). Initially, the normality of all salivary parameter data was evaluated by the Shapiro–Wilk test, followed by an analysis of the homogeneity of variance using the Levene test. Since all salivary parameters showed a deviation of normality, significant differences between the groups were evaluated using the non-parametric Kruskal–Wallis test with Dunn's post-test for multiple comparisons. Associations between clinical, sociodemographic and salivary parameters were tested through bivariate correlation and Spearman's coefficient. The significance cutoff level adopted in all analyzes was 5%.

## Results

In Table 1 is shown the demographic, anthropometric, and clinical characteristics of the volunteers enrolled in the present study. It is important to mention that none of the volunteers presented periodontal disease.

**Table 1:** Data concerning demographic, anthropometric, and clinical characteristics of the volunteers who participated in the present study

Parameters	Volunteers (*n* = 11)	
	Older women (*n* = 10)	Older man (*n* = 1)
Age (years)	67.2 ± 7.3	67
Weight (kg)	71.1 ± 9.8	72
Height (m)	1.54 ± 0.6	1.71
BMI (kg/m2)	30.1 ± 3.2	24.6
Clinical characteristics (n)		
Smoking	0	0
Ashma	0	0
COPD	0	0
Obesity	2	1
Type 2 diabetes	3	0
Hypertension	4	1

**Fig. 2:**
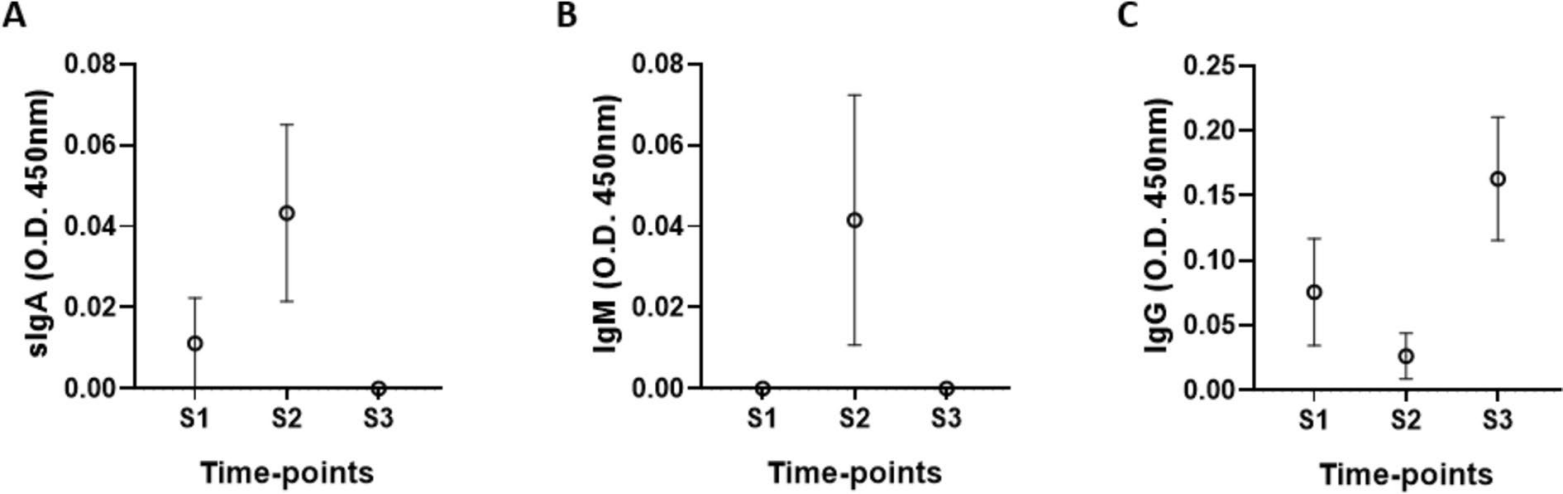
Salivary immunoglobulin levels of sIgA (**A**), IgM (**B**), and IgG (**C**) before (S1) and after 6 (S2) and 20 (S3) months of the start of the COVID-19 pandemic. Values are presented in the median and interquartile range

Even though the statistical analysis did not show a significant difference, a tendency to increase salivary levels of sIgA (Fig. 2A) and IgM (Fig. 2B) at S2 were observed. In a similar way, increasing salivary levels of IgG (Fig. 2C) were observed at S3 compared to the values found at S1 and S2. All volunteers received at least two doses of the COVID-19 vaccines after S2 and before S3 saliva sample collection.

**Fig. 3:**
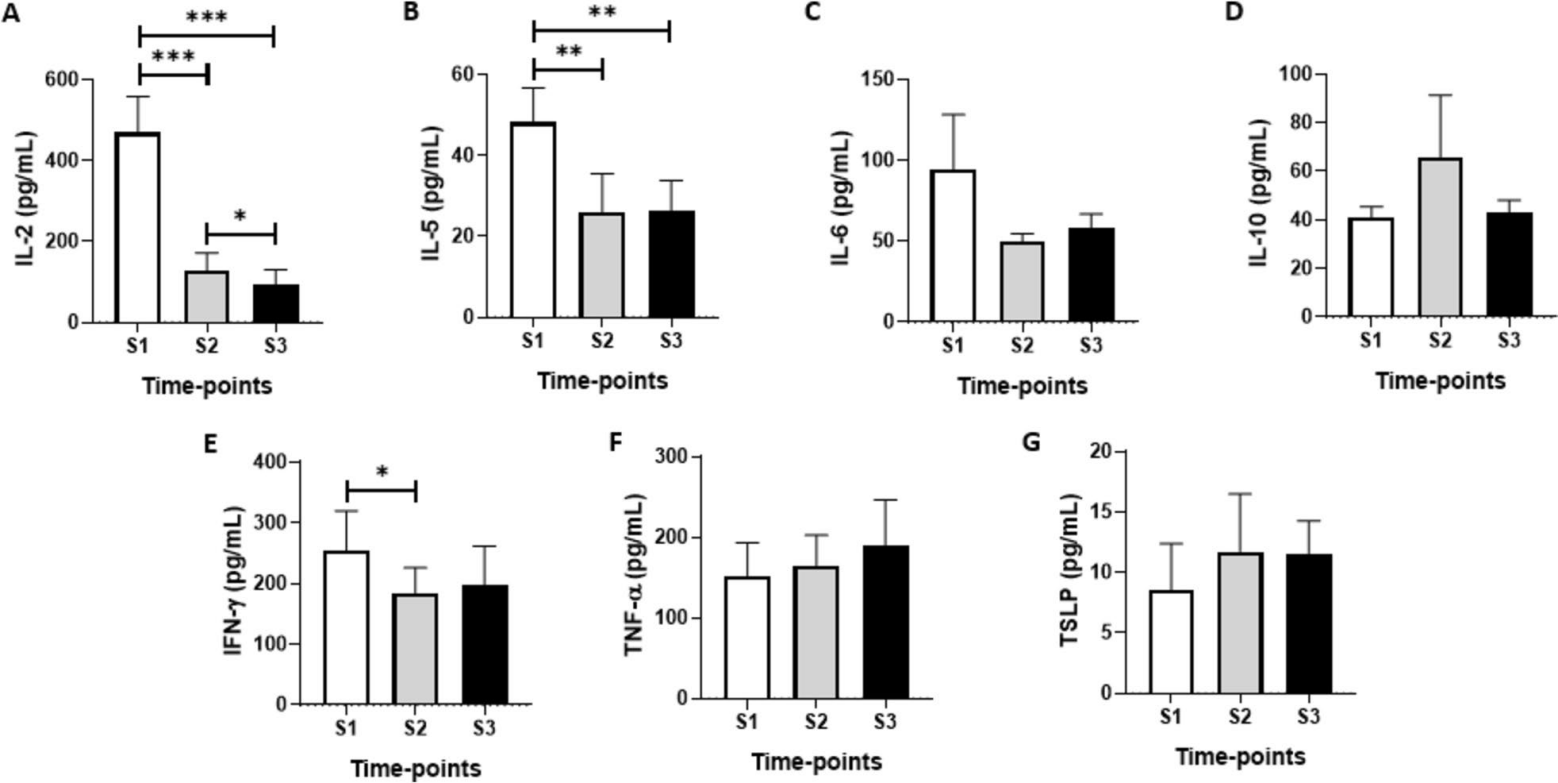
Comparison of the salivary cytokines levels of IL-2 (**A**), IL-5 (**B**), IL-6 (**C**), IL-10 (**D**), IFN-γ (**E**), TNF-α (**F**), KLOTHO (**G**) and TSLP (**H**) before (S1) and after 6 (S2) and 20 (S3) months of the start of the COVID-19 pandemic. Values are presented in the median and interquartile range. The level of significance was established at 5% (*p < 0.05; **p < 0.01; *** *p* < 0.001)

Significantly decreased levels of IL-2 (Fig. 3A) were found at S2 and S3 time points as compared to the values observed at S1 (*p* < 0.0001). Similarly, lower levels of IL-5 (Fig. 3B) were observed at S2 (*p* < 0.001) and S3 (*p* < 0.001) time points. In relation to the IFN- γ results (Fig. 3E), lower levels of this cytokine were found at S2 as compared to the values observed at S1 (*p* < 0.01). No differences were found in salivary levels of IL-6 (Fig. 3C), IL-10 (Fig. 3D), TNF-α (Fig. 3F) and TLSP (Fig. 3G) at the time points evaluated here.

**Fig. 4:**
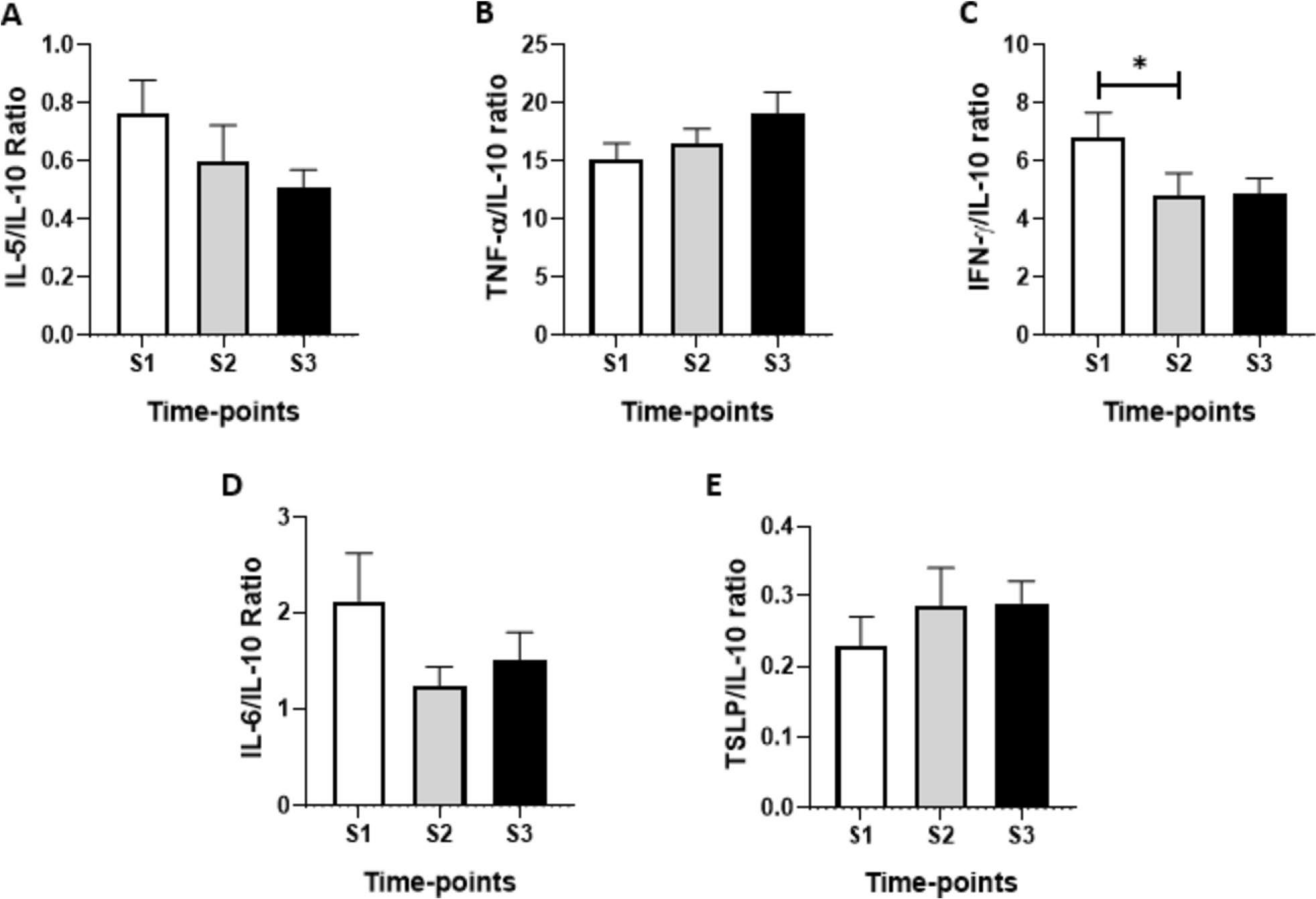
Comparison of ratios between the salivary levels of pro/anti-inflammatory cytokines IL-5/IL-10 (**A**), TNF-α/IL-10 (**B**), IFN-γ/IL-10 (**C**), IL-6/IL-10 (**D**), KLOTHO/IL-5 (**E**), KLOTHO/TNF-α (**F**), KLOTHO/INF-γ (**G**), KLOTHO/IL-6 (**H**) before (S1) and after 6 (S2) and 20 (S3) months of the start of the COVID-19 pandemic. Values are presented in the median and interquartile range. The level of significance was established at 5% (*p < 0.05)

The data obtained in the ratio analysis between the salivary levels of pro- and anti-inflammatory cytokines assessed in the present study are shown in Fig. 4. A significant decrease in the IFN- γ/IL-10 ratio (Fig. 4C) was found at S2 (*p* < 0.01).

**Fig. 5:**
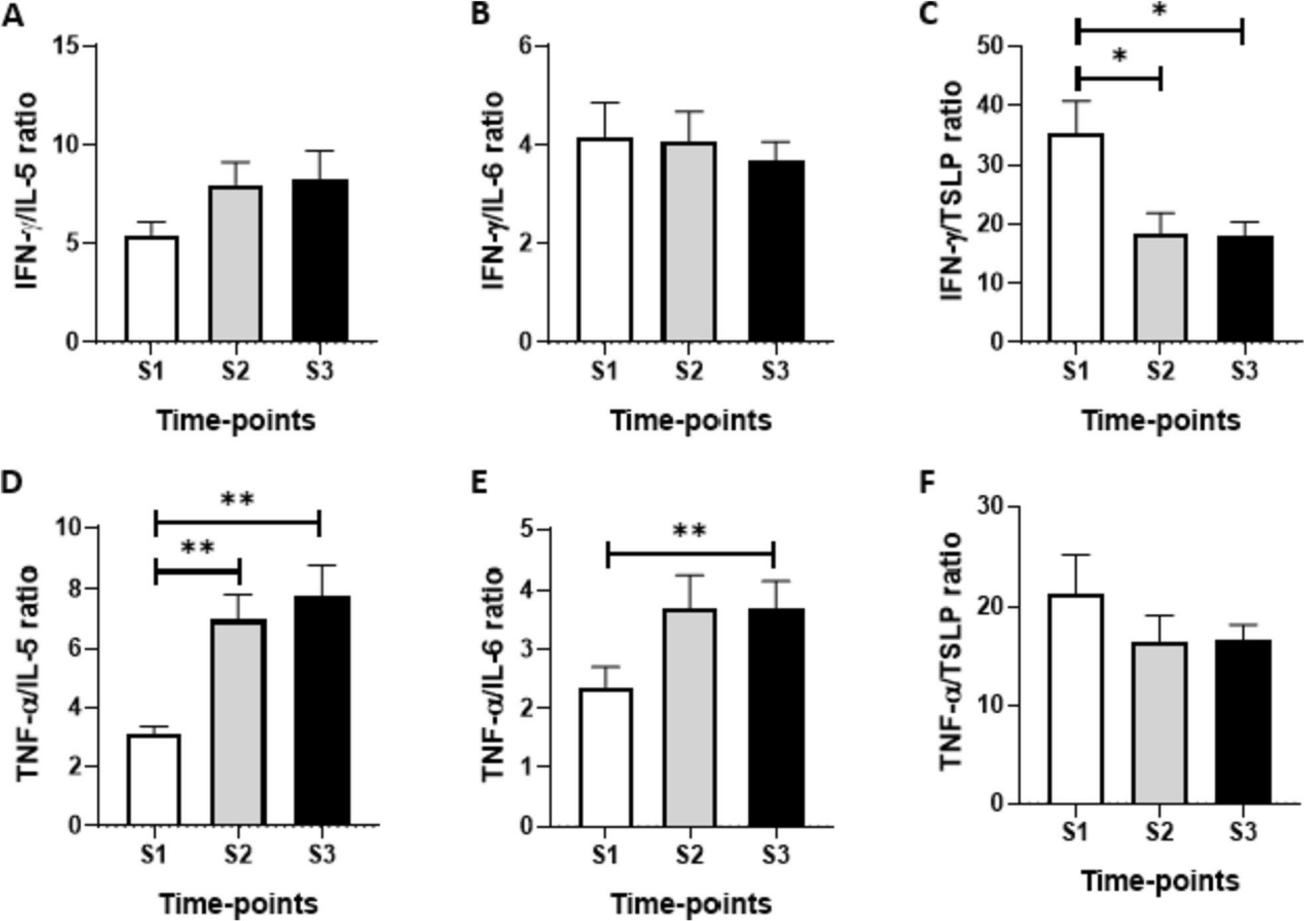
Comparison of the ratio TH1/TH2 of IFN-γ/IL-5 (**A**), TNF-α/IL-5 (**B**), INF-γ/IL-6 (**C**) and TNF-α/IL-6 (**D**) before (S1) and after 6 (S2) and 20 (S3) months of the start of the COVID-19 pandemic. Values are presented in the median and interquartile range. The level of significance was established at 5%. (**p < 0.01)

When assessing the Th1/Th2 ratios (Fig. 5), a significant decrease was found in the IFN-γ/TSLP ratio (Fig. 5C) at S2 (*p* < 0.001) and S3 (*p* < 0.001) when compared to the values at S1. In addition, a significant increase was observed in the TNF-α/ IL-5 ratio (Fig. 5D) at S2 (*p* < 0.001) and S3 (*p* < 0.001) in comparison to the values at S1. In a similar way, an increase in the TNF-α/IL-6 ratio (Fig. 5E) was observed at S3 (*p* < 0.001) when compared to the values at S1.

## Discussion

The present study provided an evaluation of the salivary levels of pro- and anti-inflammatory cytokines, as well as specific antibodies (sIgA, IgM, and IgG) for SARS-CoV-2 antigens in a group of older adults, both before and during the COVID-19 pandemic. Given the effects that social isolation has on the host, particularly concerning an immune/inflammatory dysfunction [3], here, we were able not only to present some interesting data regarding the salivary immune/inflammatory response but also to compare these findings between before and after the COVID-19 pandemic.

To the best of our knowledge, the measurement of salivary cytokines and immunoglobulins have only been assessed at the time or after coronavirus infection. Therefore, in this study, we had the opportunity to monitor immune/inflammatory parameters in a cohort of older adults via saliva in a three-point longitudinal study, which can improve our comprehension of the COVID-19 pandemic's impact on this population.

Our observations that levels of sIgA and IgM specific for SARS-CoV-2 antigens increased at the S2 time point are in agreement with the literature since the production of these antibodies in upper airways is elicited by the presence of pathogenic agents including viruses (Smith et al. 2012). In this sense, SARS-CoV-2’s presence leads to the acute production of secretory immunoglobulin A (sIgA) and secretory immunoglobulin M (sIgM) in order to improve immune protection [16–18]. Thus, the elevation of salivary levels of sIgA and sIgM specific for SARS-CoV-2 antigens was expected in a pandemic setting, even though only 2 participants reported testing positive for COVID-19, it is expected that they had contact with the virus during this period. In relation to these antibodies, it is well-known that sIgA acts as a first line of defense in the mucosa, including in the upper airways, against many types of pathogens due to its ability to inhibit the pathogen proliferation. Reductions in sIgA-levels can significantly impair the local immune response [18]. Similarly to sIgA, the presence of sIgM in the mucosa, particularly in the upper airways, is an important signal to indicate that a primary immune response was elicited by the pathogen as part of the mucosal defense against this harmful agent [16]. It is of utmost importance to also mention that the elevations of these antibody levels at the S2 time-point occurred at the time of peak infection in Brazil [19]. Regarding the elevation of IgG specific for SAR-CoV-2 antigens occurred only in the S3 time point, this finding corroborates with the classical concept of immune response after vaccination [20], since all participants reported having received at least two doses of the COVID-19 vaccine between S2 and S3 time points.

Beyond immunoglobulins, the analysis of cytokines in saliva, with both pro- and anti-inflammatory properties, is a corollary aspect to improve our understanding how the immune/inflammatory response present in upper airways could be elicited in different contexts, especially in the COVID-19 era [21].

Here, for the first time, we reported a significant decrease of salivary IL-2 levels, a cytokine that presents both mitogen properties and the ability to induce T cells differentiation to a specific regulatory profile [22], in older adults during the period with social isolation imposed by the COVID-19 pandemic (S2 and S3 time points), when compared to values found before this period (S1). Therefore, this finding allows us to suggest that lengthy social isolation (6 and 20 months) can influence the immune/inflammatory responses in this population. Corroborating these data are a handful of studies that demonstrate that chronic social stress [23], repeated restraint, and social isolation stress [24, 25] are capable of suppressing the production of IL-2, whereas other studies did not report any alteration in the levels of this cytokine in conditions of social isolation [26] or confinement [27]. Interestingly, alterations in IL-2 production in repeated restraint and isolation stress situations were associated with elevations in the levels of cortisol and ACTH, two well-known hormones released by pituitary-adrenal system in response to stress challenge [25]. In this respect, it is important to consider that many studies have suggested that social isolation also has a great impact on the mental and physical health of this population, with an increase in anxiety, depression and poor sleep quality [28, 29]. Thus, although we did not evaluate salivary cortisol levels, the pieces of information above mentioned can suggest that, together with the physical inactivity of older adults due to the social distancing recommended by the WHO, may be the factors that altered the production of IL-2 in upper airways, as observed in this study. Furthermore, it is worth mentioning that, as recently demonstrated, COVID-19 patients who presented xerostomia presented lower salivary IL-2 levels, and the authors suggested that this reduction could be related to the direct damage of SARS-CoV-2 infection on the lymphocytes sited in the salivary glands [30].

In a similar way, IL-5, a typical proinflammatory cytokine associated with Th2 immune profile [31], showed a significant decrease during the social isolation imposed by the COVID-19 pandemic (S2 and S3 time points) when compared to the values obtained before this period (S1). In the COVID-19 context, it has been reported that serum IL-5 levels can be increased [32] or not [33] in patients with severe COVID-19. Concerning the involvement of IL-5, present in upper airways, and the local response to SARS-CoV-2 infection, in a general way, studies have been focusing on patients with some diseases in the airways, mainly asthma and allergies [34–36]. Therefore, our findings that salivary IL-5 levels were significantly reduced in older adults without airway diseases-associated, in S2 and S3 time points, not only is another novelty of this study but can also putatively indicate a prominent effect of social isolation in the inflammatory status on upper airways, despite the ratio between IL-5 (a pro-inflammatory cytokine) and IL-10 (an anti-inflammatory cytokine) did not show significant alteration during this period of social isolation. Besides, it is noteworthy to mention that, even though studies aimed at evaluating the effect of stressful situations on salivary IL-5 levels are scarce, in general, these few studies demonstrate that levels of this cytokine can be acutely increased [37, 38] and fall when stress is ceased [38] or even cannot be altered by acute stress [39]. Although, in a general way, it has been shown that mental health in older people was negatively affected during the social distancing associated with the COVID-19 pandemic [29], one study showed that the social isolation in the same period turned out in a mild stressful impact [40]. Furthermore, it was verified that, during the COVID-19 quarantine, some individuals who presented lower anxiety levels also had more sleep disturbances [41]. Thus, despite the older adults enrolled in the present study being isolated in their own homes, they reported having strong social networks, which could have helped to cope with the stress during this period, as previously reported [42].

Beyond this finding that lower salivary IL-5 levels at both S2 and S3 time points than the baseline values, we also observed a significant increase in the ratio between TNF-α and IL-5 at the same time points, even though the values of TNF-α did not alter during the study time points. Based on the literature, patients with MER-CoV infection presented Th1 and Th17 responses [43]. It is broadly accepted that cytokines released in the upper airway’s mucosa as a consequence of respiratory infections can induce distinct immune responses (Th1, Th2, and Th17), which favor the generation of protective immunity, which includes antibody production in this site [44]. Although in certain occasions these immune profiles could coexist in conjunct, the classical immunological notion pointed out that, in physiologic or in some inflammation and infection situations, towards of the immune response for one immune profile, such as Th1, is associated with a diminished in the other immune profile, such as Th2 [45, 46]. Hence, the increase in the TNF-α/IL-5 ratio found ta the S2 and S3 time points is in accordance with this last information and can not only indicate that the Th1 immune profile was induced in order to improve the upper airways immunity in the study context, as well as could impact in the reduction of salivary IL-5 levels. Reinforcing this suggestion, we also observed a significant increase in the ratio between TNF-α and IL-6 (TNF-α/IL-6), another cytokine related to the Th2 immune profile, at the S3 time point. It is paramount to point out that, in terms of the function of TNF-α on upper airways immunity, it was reported that this cytokine is important to upregulate the transcription of polymeric immunoglobulin receptor (pIgR), which is a crucial factor to facilitate the transport of secretory antibodies by the epithelial cells presents in the mucosal surfaces in response to a broad spectrum of host factors or even microorganisms [47]. Based on these data, we can putatively suggest that this towards of immune profile to Th1 could influence the transport of antibodies produced by the participants enrolled in the present study through the upper airways.

In an interesting way, the salivary IFN-γ levels significantly decreased at the S2 time point compared to the baseline values (S1). In terms of IFN-γ, this type-III interferon is a pivotal cytokine involved in both innate and adaptative immune responses, especially in the immunity against viruses, which includes coronavirus infections, such as MERS, SARS-CoV, and SARS-CoV-2, due to its prominent action of inhibiting the replication of these viruses [48–51]. Corroborating these pieces of information, it was reported that the SARS-CoV-2 elimination in the upper airways is associated with IFN responses [52]. In the same way, our group showed that individuals, both asymptomatic and with mild symptoms, who presented salivary sIgA for SARS-CoV-2 also showed elevated salivary IFN-γ levels [53]. Unlike these previous data, here we observed a significant reduction of salivary IFN-γ levels at the S2 time point in comparison to the baseline values (S1), even though the antibody levels (both for sIgA and IgM) for SARS-CoV-2 were elevated in this same time point. According to the literature, beyond its remarkable action on anti-viral immunity, it has been reported that there is a close interaction between social behavior and IFN-γ levels since the elevation in social contact was related to a bolstered antiviral response, which can lead to the enhancement of the response to viral pathogen exposure [54, 55]. Moreover, it was also demonstrated a significant association between systemic IFN-γ levels and better psychosocial well-being [56]. Aligned with these data, in addition to the role of IFN-γ in the immune response to viruses’ infection, it has also been considered critical in the social behavior, which was demonstrated in mice deficiencies for IFN-γ presented severe alterations in social behavior, by showing a preference for non-social versus social stimuli [57].

In spite of the reduction of salivary IFN-γ levels at the S2 time point, which could lead to a decrease in the immune response to SARS-CoV-2 infection, the lower ratio between IFN-γ /IL-10 observed at the same time point, a consequence of both reduction of IFN-γ and a tendency to elevation in IL-10 levels, is an important finding since demonstrates that a pivotal control the local inflammation was elicited, which already was formerly documented by our group [53]. Furthermore, this regulatory process not only can putatively favor the production and release of antibodies in the upper airways but also could contribute to the participants being asymptomatic for COVID-19. In agreement with the literature, the ratio between pro and anti-inflammatory cytokines is useful and crucial to verify whether the inflammatory process elicited by infection has been controlled [58], and also allows us to better elucidate the occurrence of a dysregulated inflammatory response, such as the cytokine storm, during the SARS-CoV-2 infection [59].

In terms of generating a favorable environment for antibody production on airway mucosa, it is important to cite the effect of TSLP, a cytokine produced by the tonsillar crypt epithelium, which can promote the class switch of IgA for type 1, the more important IgA in the mucosa [16]. TSLP is produced and released after contact with external agents such as viruses that target the airways mucosa, in order to improve the adaptative immune response against this pathogenic agent [60]. However, in higher amounts, this cytokine is released into the bloodstream, and it is closely associated with exacerbated immune responses, particularly in those involved in the establishment of allergy and asthma [61] since this molecule is a promotor of Th2 immune responses [32]. Increased TSLP levels are found in COVID-19 patients and have been associated with the severity of this disease [32]. Based on this finding, the use of TSLP inhibitors has been suggested to control severe lung inflammation in COVID-19 [62]. In the present study, although the salivary TSLP levels did not show significant differences in the time points assessed here, the tendency of elevation in their levels, observed at S2 and S3 time points, even though not significant could influence the reduction of salivary IFN-γ levels, which was evidenced in the lower IFN-γ/TSLP ratio found at the S2 and S3 time points when compared to the S1 values.

Therefore, the salivary inflammatory status observed in the present study allows us putatively to suggest that a favorable immune/inflammatory response both against the virus infection and vaccination was elicited in the upper airways in the older adult population participants of this study.

Although a limitation of the study can be attributed to the small number of participants and a convenience sample, it is of utmost importance to point out that: 1) due to the social isolation imposed by the COVID-19 pandemic it was difficult to recruit a large number of older adults to participate in this study, and 2) the main results found in this study, mainly in terms of salivary levels of cytokines, were obtained by comparing the data of each participant both before and after the COVID-19 pandemic, in duplicate analysis, which can mitigate this drawback.

## Conclusions

Taken together, the results presented in this study add significant new information concerning the effect of social isolation imposed by COVID-19 on the immune/inflammatory responses in the mucosa of the upper airways, particularly those present in the oral cavity, in the older adult population, by showing that a controlled deviation of Th1 and Th2 immune responses, both during infection and after vaccination, can generate favorable conditions to combat this viral infection without exacerbation of the immune response, and aggravation of the pathology. Lastly, our data also reinforce the value of saliva for monitoring the immune/inflammatory response in older adults.

## Data Availability

All data generated or analysed during this study are included in this published article.
